# Supplementary material for: A Network Toxicology Approach for Mechanistic Modelling of Nanomaterial Hazard and Adverse Outcomes
Source: Adv Sci (Weinh). 2024 Jun 25;11(32):2400389. doi: 10.1002/advs.202400389 (PMC11348149; doi:10.1002/advs.202400389)
Supplement: Supplementary file 1 — Supporting Information [file ADVS-11-2400389-s001.pdf]

ADVANCED SCIENCE
Open Access

Supporting Information

for *Adv. Sci.*, DOI 10.1002/advs.202400389

A Network Toxicology Approach for Mechanistic Modelling of Nanomaterial Hazard and Adverse Outcomes

*Giusy del Giudice, Angela Serra, Alisa Pavel, Marcella Torres Maia, Laura Aliisa Saarimäki, Michele Fratello, Antonio Federico, Harri Alenius, Bengt Fadeel and Dario Greco**

Supporting Information

A Network Toxicology Approach for Mechanistic Modelling of Nanomaterial Hazard and Adverse Outcomes

Giusy del Giudice , Angela Serra , Alisa Pavel , Marcella Torres Maia, Laura Aliisa Saarimäki, Michele Fratello , Antonio Federico , Harri Alenius , Bengt Fadeel , Dario Greco.

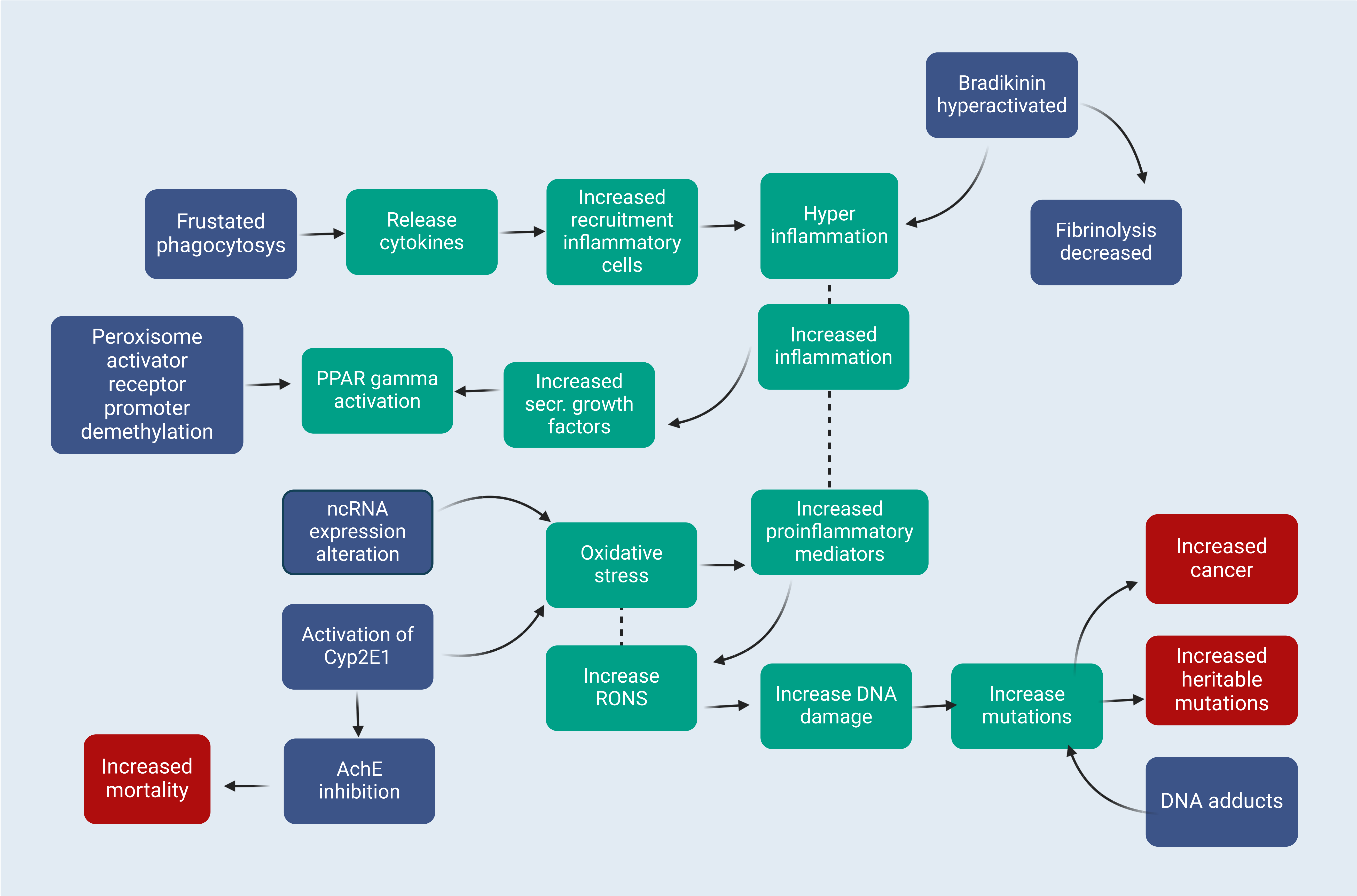

Figure S1: Reconstructed mechanism of response to MWCNT exposure.

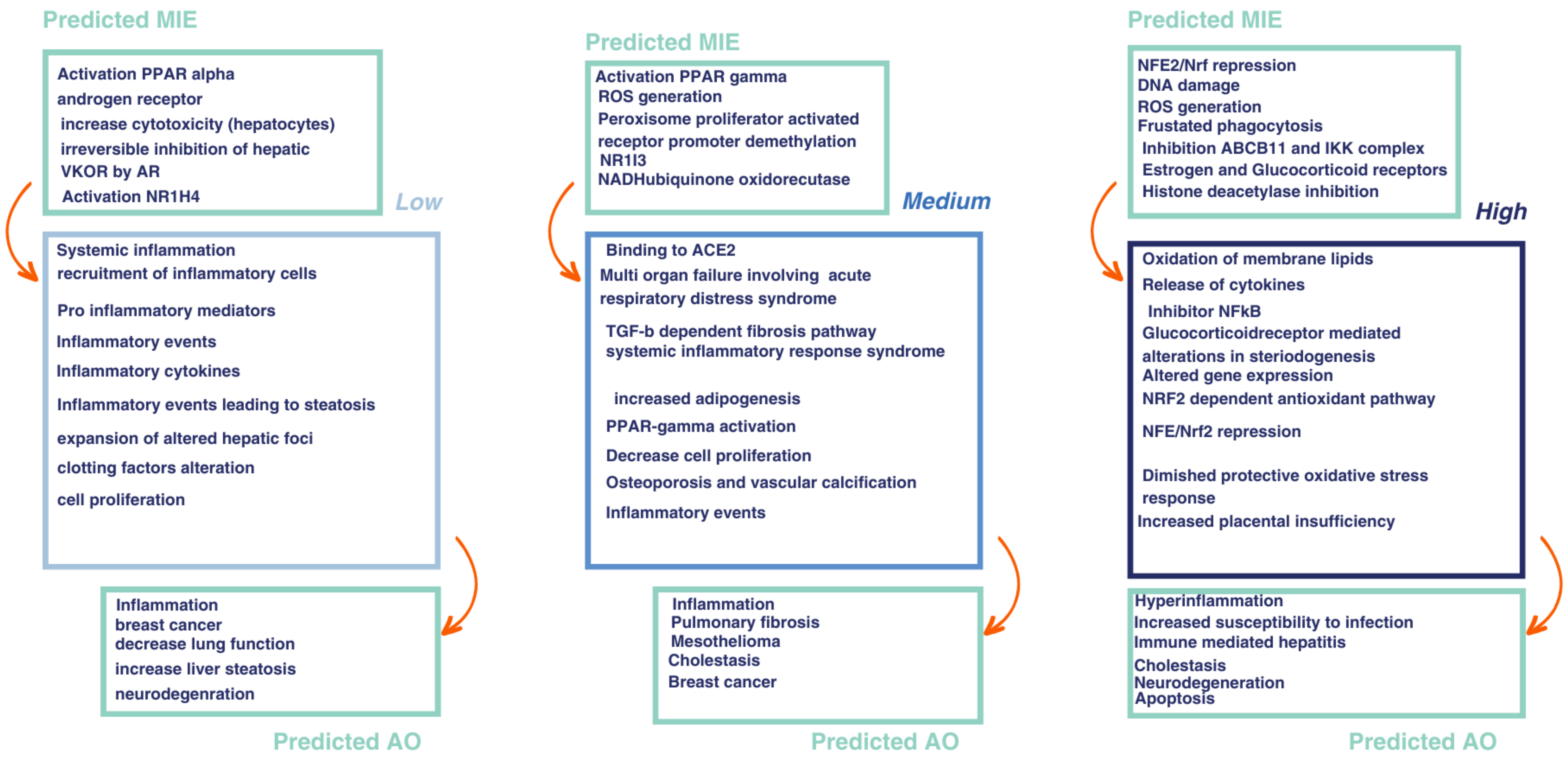

Figure S2: AOP-based reconstructed mechanism of materials inducing different levels of neutrophil infiltration, as described in Fortino et al. In this case, co-expression network representations of the exposures were used as the input of the framework.

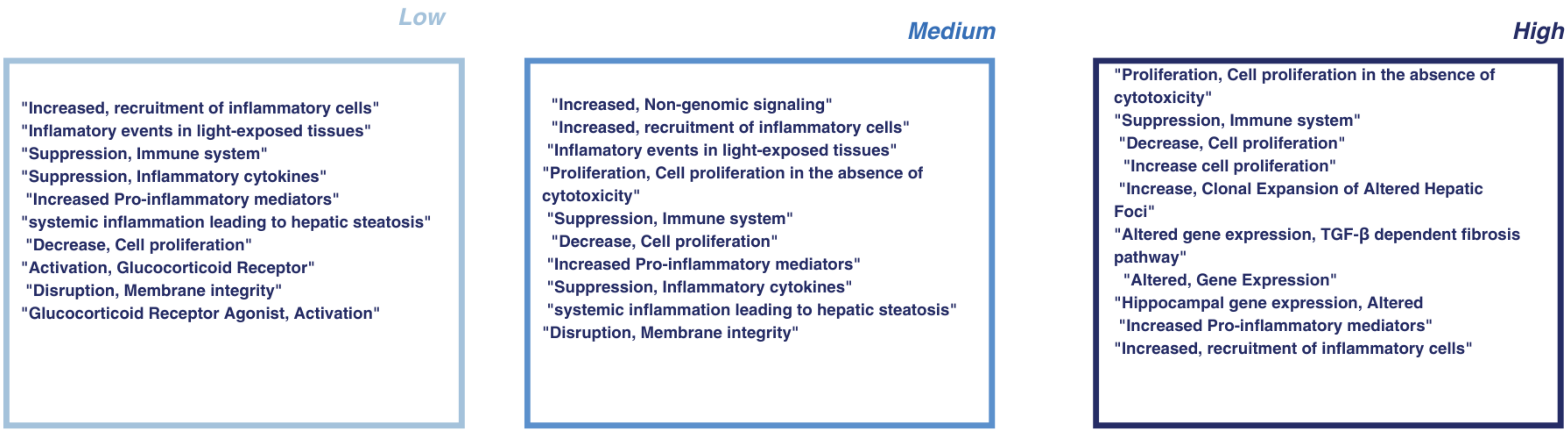

Figure S3: AOP-based reconstructed mechanism of materials inducing different levels of neutrophil infiltration, as described in Fortino et al. In this case, lists of differentially expressed genes of the exposures were used as the input of the framework.

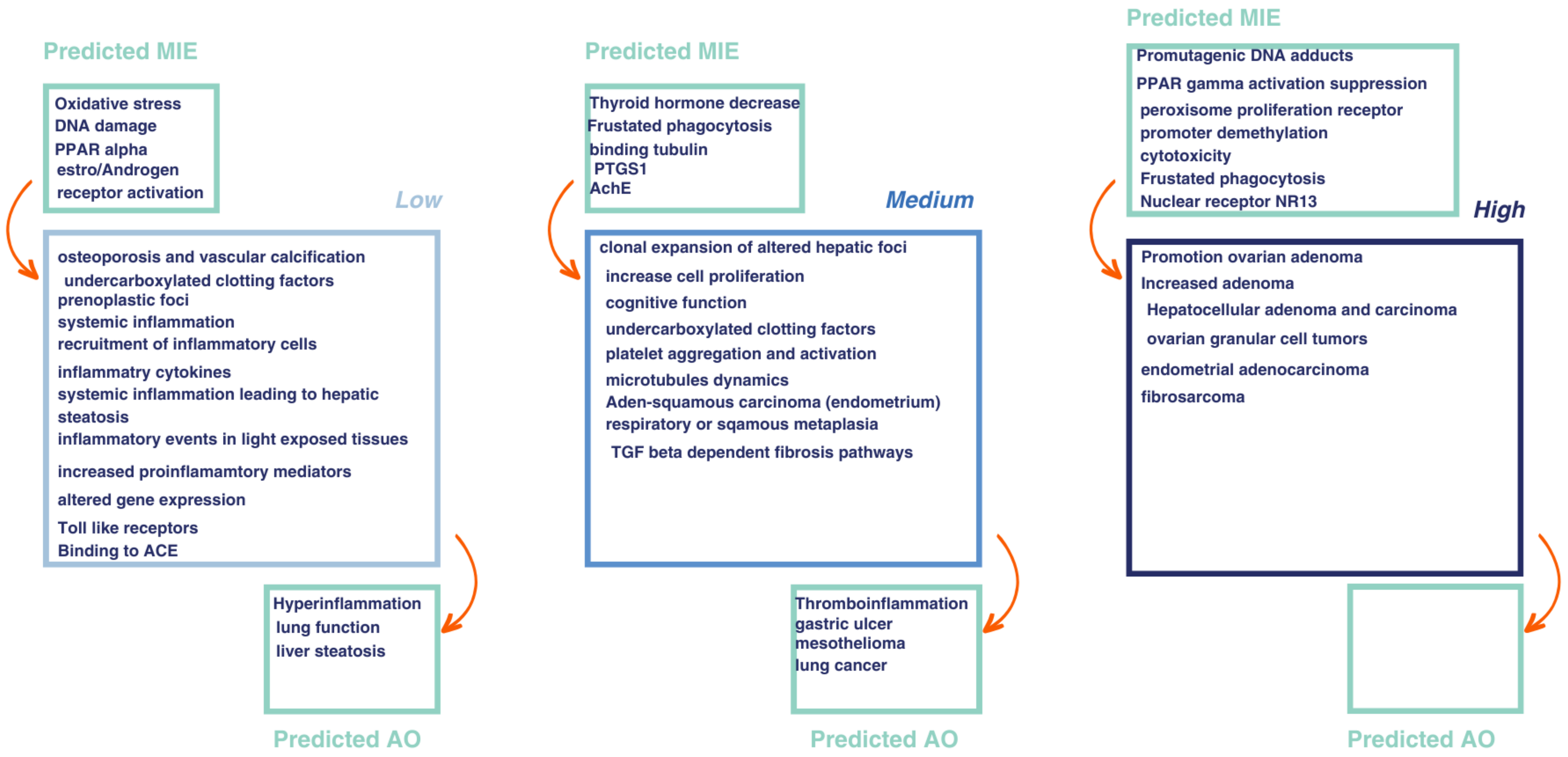

Figure S4: AOP-based reconstructed mechanism of materials associated with different hazard levels, as described in Fortino et al.

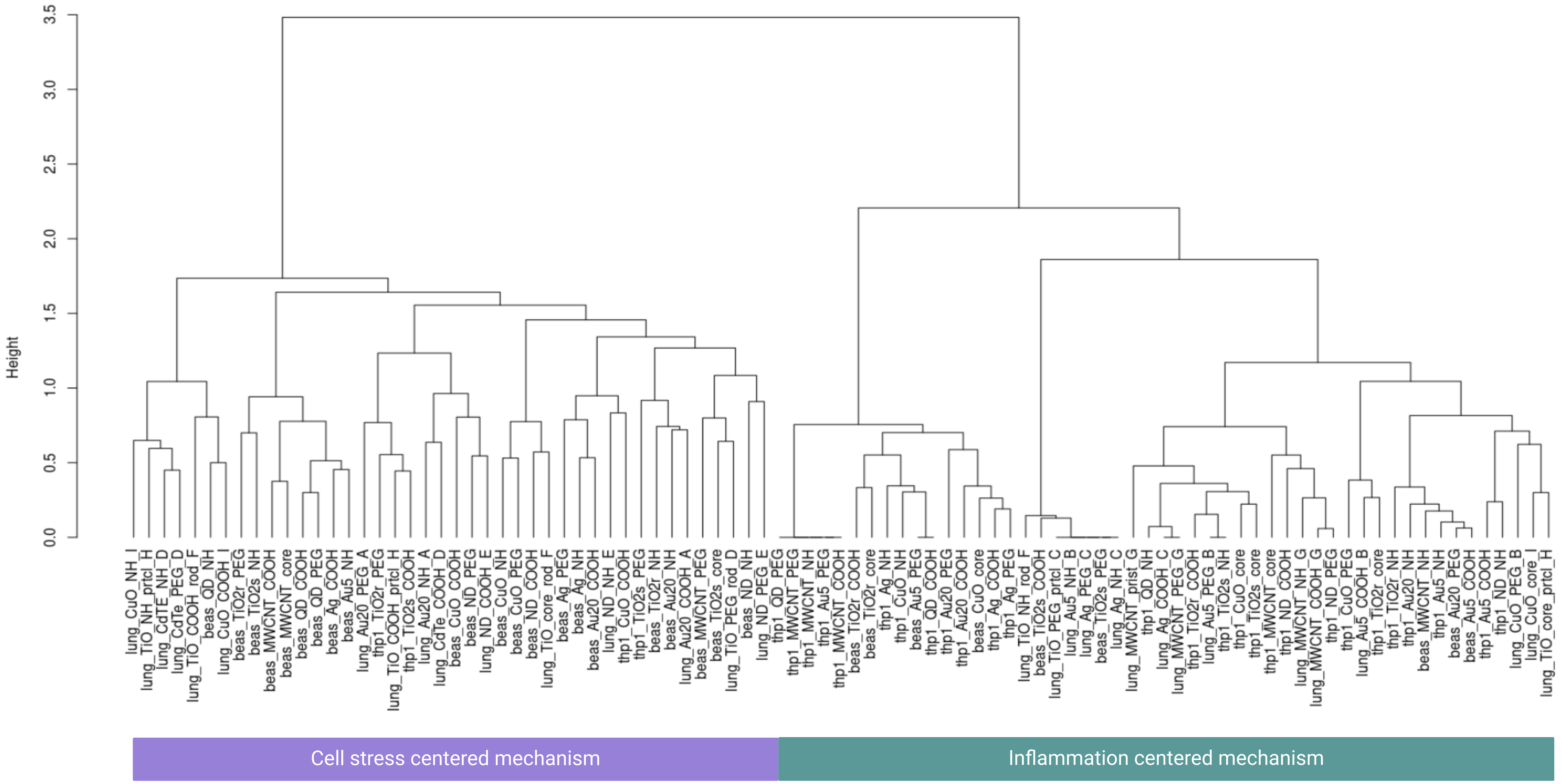

Figure S5: Dendogram representing the hierarchical clustering of the 93 exposure AOP-based responses.The distance used is based on the mean of the jaccard index between networks nodes and edges Annotation has been prerformed evaluating the results of the AOP-based mechanism for each exposure.

Table S1: Enriched events in the three test systems (CuO_Core)

THP1	Lung
systemic inflammatory response syndrome	Increased, inflammation
Diminished protective oxidative stress response	Suppression, Inflammatory cytokines
Increased, recruitment of inflammatory cells	systemic inflammatory response syndrome
Decrease, Cell proliferation	Increased, recruitment of inflammatory cells
Increased, inflammation	Inflamatory events in light-exposed tissues
systemic inflammation leading to hepatic steatosis	Increased Pro-inflammatory mediators
Inflamatory events in light-exposed tissues	systemic inflammation leading to hepatic steatosis
Increased Pro-inflammatory mediators	Altered, Chromosome number
NFE2/Nrf2 repression	Increased, Chromosome misseggregation
Suppression, Inflammatory cytokines	Disruption, Microtubule dynamics
Altered gene expression, NRF2 dependent antioxidant pathway	Binding of microtubule stabilizing agents (MSA) to microtubules
Decreased, Apoptosis (Epithelial Cells)	Impaired inguinoscrotal testicular descent phase

BEAS-2B
Altered, Gene Expression
Decrease, Cell proliferation
systemic inflammatory response syndrome
Antagonism, Thyroid Receptor
Increase cell proliferation
Increase, Clonal Expansion of Altered Hepatic Foci
Increased, recruitment of inflammatory cells
Increased, inflammation
Apoptotic cell death
Inflamatory events in light-exposed tissues
systemic inflammation leading to hepatic steatosis
Suppression, Inflammatory cytokines
Increased Pro-inflammatory mediators
Altered gene expression, TGF-β dependent fibrosis pathway
Binding to ACE2
Increase activation, Nuclear factor kappa B (NF-kB)
Release, Cytokine

THP1-AO predicted	Lung-AO predicted
Inflamatory events in light-exposed tissues	Inflamatory events in light-exposed tissues
Decrease, Growth	Hyperinflammation
Increased, mesotheliomas	N/A, Breast Cancer
Hyperinflammation	Increased, mesotheliomas
Increased, Ductal Hyperplasia	N/A, Neurodegeneration
N/A, Neurodegeneration	Reduced, Reproductive Success
Increase, DNA damage	Malformation, cryptorchidism - maldescended testis
Increased, Liver Steatosis	Increased, Liver Steatosis
Increased, steatosis	Increase, Aneuploid offspring
Ororofacial clefting	Sensory axonal peripheral neuropathy
Apoptotic cell death	Increase, Mutations
N/A, Breast Cancer	N/A, Liver fibrosis
N/A, Liver fibrosis	Decrease, Lung function
Increase, Mutations	Accumulation, Liver lipid
Decrease, Lung function	impaired, Fertility
Accumulation, Liver lipid	Impairment, Learning and memory
Increased Mortality	Increased Mortality
impaired, Fertility	

BEAS-2B-AO predicted
Inflamatory events in light-exposed tissues
Decrease, Growth
Apoptotic cell death
Hyperinflammation
Increased, mesotheliomas
Ororofacial clefting
Cognitive Function, Decreased
Neural tube defects
Cholestasis, Pathology
N/A, Neurodegeneration
Immune mediated hepatitis
N/A, Breast Cancer
Increased, Liver Steatosis
Increase, Mutations
Increased, Ductal Hyperplasia
Decrease, Lung function
Increased Mortality

THP1-MIE predicted	Lung-MIE predicted
systemic inflammation leading to hepatic steatosis	systemic inflammation leading to hepatic steatosis
NFE2/Nrf2 repression	Binding of microtubule stabilizing agents (MSA) to microtubules
Alkylation, Protein	Oxidative Stress
Bradykinin system, hyperactivated	Binding, Tubulin
Increase, DNA damage	Bradykinin system, hyperactivated
ROS generation from photoactivated chemicals	Alkylation, Protein
Covalent Binding, Protein	Covalent Binding, Protein
Oxidative Stress	Increase, DNA damage
Fibrinolysis, decreased	ROS generation from photoactivated chemicals
Activation, Glucocorticoid Receptor	Frustrated phagoytosis
Binding, Thiol/seleno-proteins involved in protection against oxida	Activation, Glucocorticoid Receptor
Frustrated phagoytosis	Activation of Cyp2E1
Increase in reactive oxygen and nitrogen species (RONS)	Antagonism, Androgen receptor
Activation of Cyp2E1	Fibrinolysis, decreased
Inhibition, IKK complex	Increase in reactive oxygen and nitrogen species (RONS)
Non-coding RNA expression profile alteration	Inhibition, IKK complex
Inhibition, NADH-ubiquinone oxidoreductase (complex I)	Non-coding RNA expression profile alteration
Acetylcholinesterase (AchE) Inhibition	Inhibition, NADH-ubiquinone oxidoreductase (complex I)

BEAS-2B-MIE predicted
systemic inflammation leading to hepatic steatosis
Antagonism, Thyroid Receptor
Oxidative Stress
Alkylation, Protein
Inhibition, Bile Salt Export Pump (ABCB11)
Increase, Cytotoxicity (epithelial cells)
Histone deacetylase inhibition
Frustrated phagoytosis
Activation, Estrogen receptor
benzoquinone imine and acylglucuronide metabolites
ROS generation from photoactivated chemicals
Activation of Cyp2E1
Increase, DNA damage
Covalent Binding, Protein
Increase in reactive oxygen and nitrogen species (RONS)
Inhibition, IKK complex

Enriched events in the three test systems (CuO_NH2)

THP1	Lung	BEAS-2B
systemic inflammatory response syndrome	Altered, Chromosome number	Cognitive Function, Decreased
Decrease, Cell proliferation	Increased, Chromosome misseggregation	Altered, Gene Expression
Increased, recruitment of inflammatory cells	Increase, Clonal Expansion of Altered Hepatic Foci	Apoptotic cell death
Inflamatory events in light-exposed tissues	Increase cell proliferation	General Apoptosis
Increased, inflammation	Disruption, Microtubule dynamics	Increase, Apoptosis
Increase activation, Nuclear factor kappa B (NF-kB)	Decrease, Cell proliferation	Decreased, Apoptosis (Epithelial Cells)
Increased Pro-inflammatory mediators	Altered, Meiotic chromosome dynamics	Apoptosis of adult Leydig cells, Decreased testosterone by adult Leydig cells
systemic inflammation leading to hepatic steatosis	Binding of microtubule stabilizing agents (MSA) to microtubules	Apoptosis
Suppression, Inflammatory cytokines	Suppression, Inflammatory cytokines	Antagonism, Thyroid Receptor
Inhibition, Nuclear factor kappa B (NF-kB)	Inadequate DNA repair	Abnormal, Glucose homeostasis
Apoptotic cell death	Disorganization, Meiotic Spindle	Decreased steroidogenesis, Increased Apoptosis of Adult Leydig Cells
Diminished protective oxidative stress response	systemic inflammation leading to hepatic steatosis	Cell injury/death
Increase, Clonal Expansion of Altered Hepatic Foci		persistent, cytotoxicity (pleura or peritoneum)
Increase cell proliferation		
Altered gene expression, TGF-β dependent fibrosis pathway		
Decreased, Apoptosis (Epithelial Cells)		

THP1-AO predicted	Lung-AO predicted	BEAS-2B-AO predicted
Inflamatory events in light-exposed tissues	Reduced, Reproductive Success	Apoptosis
Increase, Increased susceptibility to infection	Decrease, Growth	Apoptotic cell death
Decrease, Growth	Increased, Liver Steatosis	N/A, Neurodegeneration
Increased, mesotheliomas	Ororofacial clefting	Decrease, Growth
Apoptotic cell death	Increase, Aneuploid offspring	Neurodegeneration
Hyperinflammation	Sensory axonal peripheral neuropathy	Gestational diabetes mellitus
Increased, Ductal Hyperplasia	Accumulation, Liver lipid	Reduce, Sperm count
Increased, Liver Steatosis	Impairment, Learning and memory	Neural tube defects
Immune mediated hepatitis	impaired, Fertility	Cognitive Function, Decreased
Ororofacial clefting	Increased Mortality	Increase risk, microcephaly
N/A, Neurodegeneration	N/A, Liver fibrosis	Liver Injury
N/A, Breast Cancer		Increased, Ductal Hyperplasia
Increase, Mutations		Increase, DNA damage
Decrease, Lung function		Necrotic Tissue
Increased Mortality		Immune mediated hepatitis
		N/A, Breast Cancer
		impaired, Fertility

THP1-MIE predicted	Lung-MIE predicted	BEAS-2B-MIE predicted
systemic inflammation leading to hepatic steatosis	systemic inflammation leading to hepatic steatosis	Oxidative Stress
Oxidative Stress	Binding of microtubule stabilizing agents (MSA) to microtubules	Antagonism, Thyroid Receptor
Activation, Glucocorticoid Receptor	Increase, DNA damage	Alkylation, Protein
Increase, Cytotoxicity (epithelial cells)	Binding, Tubulin	Mitochondrial impairment
Bradykinin system, hyperactivated	Histone deacetylase inhibition	Histone deacetylase inhibition
Alkylation, Protein	Activation, Glucocorticoid Receptor	Activation, Estrogen receptor
Increase, DNA damage	Inhibition, IKK complex	Increased, Hepatic thyroid hormone uptake/transport
ROS generation from photoactivated chemicals	Oxidative Stress	Activation, AhR
Covalent Binding, Protein	Inhibition, NADH-ubiquinone oxidoreductase (complex I)	Thyroperoxidase, Inhibition
Prolonged TLR9 activation	Binding of inhibitor to mitochondrial complex III	Overactivation, NMDARs
Inhibition, IKK complex	Increase, Cytotoxicity (epithelial cells)	Increase, DNA damage
Activation of Cyp2E1	Increase in reactive oxygen and nitrogen species (RONS)	Inhibition, NADH-ubiquinone oxidoreductase (complex I)
Frustrated phagoytosis	Overactivation, NMDARs	Non-coding RNA expression profile alteration
Fibrinolysis, decreased	Activation, PARP1	Thyroid hormone synthesis, Decreased
benzoquinone imine and acylglucuronide metabolites	Binding of inhibitor, NADH-ubiquinone oxidoreductase (complex I)	Inhibition, Na+/I- symporter (NIS)
Increase in reactive oxygen and nitrogen species (RONS)		Acetylcholinesterase (AchE) Inhibition
Oxidation, Glutathione (To be considered with MIE)		
Binding of agonist, Angiotensin II receptor type 1 receptor (AT1R)		

Enriched events in the three test systems (MWCNT)

THP1	Lung	BEAS-2B
systemic inflammatory response syndrome	Increased, inflammation	Altered, Gene Expression
Increased, recruitment of inflammatory cells	Increased, recruitment of inflammatory cells	Increase, Preneoplastic foci (hepatocytes)
Increased Pro-inflammatory mediators	systemic inflammatory response syndrome	modulation, Extracellular Matrix Composition
Suppression, Inflammatory cytokines	Bulky DNA adducts, increase	sensitisation, skin
systemic inflammation leading to hepatic steatosis	Release, Cytokine	Increase, hepatocellular adenomas and carcinomas
Increased, inflammation	Increased Pro-inflammatory mediators	Increased, Firbrosarcoma
Inflamatory events in light-exposed tissues	Increased, secretion of proinflammatory mediators	Tumorigenesis, Hepatocellular carcinoma
Increased, Intracellular Calcium overload	Lung fibrosis	Increase, Endometrial adenocarcinomas
Cognitive Function, Decreased	Increased, secretion of local growth factors	Altered gene expression, TGF-β dependent fibrosis pathway
Increased, Angiogenesis	Impaired inguinoscrotal testicular descent phase	Increased, adenomas (pituitary)
Binding to ACE2	Inflamatory events in light-exposed tissues	Testicular Cancer
		Promotion, ovarian granular cell tumors
		Increase, lung cancer

THP1-AO predicted	Lung-AO predicted	BEAS-2B-AO predicted
Inflamatory events in light-exposed tissues	Inflamatory events in light-exposed tissues	sensitisation, skin
Lung fibrosis	Lung fibrosis	Tumorigenesis, Hepatocellular carcinoma
Cognitive Function, Decreased	Hyperinflammation	Increase, Endometrial adenocarcinomas
Increased, mesotheliomas	Increased, mesotheliomas	Increased, adenomas (pituitary)
Hyperinflammation	N/A, Breast Cancer	Promotion, ovarian granular cell tumors
N/A, Breast Cancer	Malformation, cryptorchidism - maldescended testis	Increase, lung cancer
N/A, Neurodegeneration	Cholestasis, Pathology	Neural tube defects
N/A, Liver fibrosis	N/A, Neurodegeneration	Increased, Ductal Hyperplasia
Increase, Mutations	Increase, Mutations	Increase, DNA damage
Increased, Liver Steatosis	Increase, Cancer	
Decrease, Growth	Increase, Heritable mutations in offspring	
Necrotic Tissue	Decrease, Lung function	
Decrease, Lung function	Increased Mortality	
Accumulation, Liver lipid		
Increased Mortality		

THP1-MIE predicted	Lung-MIE predicted	BEAS-2B-MIE predicted
systemic inflammation leading to hepatic steatosis	Bulky DNA adducts, increase	Formation, Pro-mutagenic DNA Adducts
ROS generation from photoactivated chemicals	Frustrated phagoytosis	Activation, Androgen receptor
Antagonism, Thyroid Receptor	Activation of specific nuclear receptors, PPAR-gamma activation	Activation, Constitutive androstane receptor
Oxidative Stress	Covalent Binding, Protein	Increase, Cytotoxicity (hepatocytes)
Inositol triphosphate receptor activation	Fibrinolysis, decreased	Activation, Estrogen receptor
Overactivation, NMDARs	Oxidative Stress	Histone deacetylase inhibition
Covalent Binding, Protein	Inhibition, Bile Salt Export Pump (ABCB11)	Alkylation, DNA
Alkylation, Protein	Increase, DNA damage	Activation, PPARα
Thyroperoxidase, Inhibition	peroxisome proliferator activated receptor promoter demethylation	Increase, DNA damage
Activation, AhR	Alkylation, Protein	Covalent Binding, Protein
Increased, Hepatic thyroid hormone uptake/transport	ROS generation from photoactivated chemicals	Activation of specific nuclear receptors, PPAR-gamma activation
Frustrated phagoytosis	Bradykinin system, hyperactivated	Increase, Oxidative damage to DNA
Thyroid hormone synthesis, Decreased	Antagonism, Androgen receptor	Bulky DNA adducts, increase
Binding of agonist, Ionotropic glutamate receptors	Increase in reactive oxygen and nitrogen species (RONS)	Increased, DNA damage and mutation
Increase, DNA damage	Activation of Cyp2E1	Oxidative Stress
Acetylcholinesterase (AchE) Inhibition	Non-coding RNA expression profile alteration	Activation, NR1H4
Inhibition, Na+/I- symporter (NIS)	Inhibition, NADH-ubiquinone oxidoreductase (complex I)	Increase in reactive oxygen and nitrogen species (RONS)
Increase in reactive oxygen and nitrogen species (RONS)	Acetylcholinesterase (AchE) Inhibition	
Activation of Cyp2E1		
Inhibition, IKK complex		

Enriched events in the three test systems

THP1	Lung	BEAS-2B
Decrease, Cell proliferation	Altered, Chromosome number	Tumorigenesis, Hepatocellular carcinoma
Increase, Preneoplastic foci (hepatocytes)	Increased, Chromosome misseggregation	Increase, hepatocellular adenomas and carcinomas
Increased CGRP, neuronal release of CGRP	Increase cell proliferation	Testicular Cancer
Increased Pro-inflammatory mediators	Increase, Clonal Expansion of Altered Hepatic Foci	Increased, Firbrosarcoma
systemic inflammation leading to hepatic steatosis	Suppression, Inflammatory cytokines	Increased, adenomas (pituitary)
Increased, inflammation	systemic inflammation leading to hepatic steatosis	Increase, lung cancer
systemic inflammatory response syndrome	Increased, inflammation	Promotion, ovarian adenomas
Increased, recruitment of inflammatory cells	Altered, Meiotic chromosome dynamics	Increase, Endometrial adenocarcinomas
Suppression, Inflammatory cytokines	Increased, recruitment of inflammatory cells	Increase, Respiratory or Squamous Metaplasia
	Increased Pro-inflammatory mediators	Promotion, ovarian granular cell tumors
	systemic inflammatory response syndrome	Increased, adenosquamous carcinomas of endometrium
	Inflamatory events in light-exposed tissues (TiO2s)	modulation, Extracellular Matrix Composition
		Increased, Intracellular Calcium overload
		Glucocorticoid Receptor mediated alterations in steriodogenic enzymes, Decreased testosterone by adult Leydig cells

THP1-AO predicted	Lung-AO predicted	BEAS-2B-AO predicted
Hyperinflammation	Inflamatory events in light-exposed tissues	Tumorigenesis, Hepatocellular carcinoma
Decrease, Growth	Hyperinflammation	Increase, Endometrial adenocarcinomas
Increased, mesotheliomas	Reduced, Reproductive Success	Promotion, ovarian adenomas
Increased Respiratory irritability and Chronic Cough,	N/A, Breast Cancer	Promotion, ovarian granular cell tumors
N/A, Neurodegeneration	N/A, Neurodegeneration	Increased, adenosquamous carcinomas of endometrium
N/A, Breast Cancer	Increase, Aneuploid offspring	Increased, adenomas (pituitary)
Ororofacial clefting	Increased, mesotheliomas	Increase, lung cancer
N/A, Liver fibrosis	impaired, Fertility	N/A, Neurodegeneration
Trigeminal and/or vagal nerve excitation causes Airway Hyper-respon	Increased, Liver Steatosis	Decrease, Growth
Increased, Liver Steatosis	N/A, Liver fibrosis	Increase, Site of Contact Nasal Tumors
Increase, Mutations	Increase, Mutations	Increased, mesotheliomas
Decrease, Lung function	Decrease, Lung function	Necrotic Tissue
Accumulation, Liver lipid	Accumulation, Liver lipid	
Increased Mortality	Increased Mortality	

THP1-MIE predicted	Lung-MIE predicted	BEAS-2B-MIE predicted
systemic inflammation leading to hepatic steatosis	systemic inflammation leading to hepatic steatosis	Formation, Pro-mutagenic DNA Adducts
Increase, Cytotoxicity (hepatocytes)	Binding, Tubulin	Inositol triphosphate receptor activation
Activation, Androgen receptor	Oxidative Stress	Overactivation, NMDARs
Activation, PPARα	Activation, Glucocorticoid Receptor	Binding of agonist, Ionotropic glutamate receptors
Alkylation, Protein	Alkylation, Protein	Alkylation, DNA
Covalent Binding, Protein	Activation of Cyp2E1	Increase, DNA damage
Oxidative Stress	Frustrated phagoytosis	Increased, DNA damage and mutation
Activation of Cyp2E1	Histone deacetylase inhibition	Increase, Oxidative damage to DNA
Bradykinin system, hyperactivated	Bradykinin system, hyperactivated	Bulky DNA adducts, increase
Increase, DNA damage	ROS generation from photoactivated chemicals	Activation of metabotropic glutamate receptor
Frustrated phagoytosis	Increase, DNA damage	Activation of specific nuclear receptors, PPAR-gamma activation
Fibrinolysis, decreased	Covalent Binding, Protein	Decrease, Intracellular pH
Activation, Glucocorticoid Receptor	Fibrinolysis, decreased	Inhibition GABAA receptor
Increase in reactive oxygen and nitrogen species (RONS)	Increase in reactive oxygen and nitrogen species (RONS)	Oxidative Stress
Inhibition, IKK complex	Inhibition, IKK complex	Increase, Cytotoxicity (epithelial cells)
Inhibition, NADH-ubiquinone oxidoreductase (complex I)	Inhibition, NADH-ubiquinone oxidoreductase (complex I)	Peptide Oxidation
Non-coding RNA expression profile alteration	Non-coding RNA expression profile alteration	Inhibition, NADH-ubiquinone oxidoreductase (complex I)
Acetylcholinesterase (AchE) Inhibition		Activated, presynaptic neuron 1
		peroxisome proliferator activated receptor promoter demethylation
		Increase in reactive oxygen and nitrogen species (RONS)

Enriched events in the three test systems (TiO2r)

THP1	Lung	BEAS-2B
Altered, Gene Expression	Cognitive Function, Decreased	Increase, Clonal Expansion of Altered Hepatic Foci
Release, Cytokine	Increased, adenomas (pituitary)	Increase cell proliferation
Increase cell proliferation	Oxidation of membrane lipids	Altered, Gene Expression
Increased Pro-inflammatory mediators	Increased, adenosquamous carcinomas of endometrium	Decrease, Cell proliferation
Altered gene expression, TGF-β dependent fibrosis pathway	Increase, Respiratory or Squamous Metaplasia	systemic inflammatory response syndrome
systemic inflammation leading to hepatic steatosis	Activation, AKT2	Increased, recruitment of inflammatory cells
modulation, Extracellular Matrix Composition	Increase, Preneoplastic foci (hepatocytes)	Increased, inflammation
Suppression, Inflammatory cytokines	Endocytotic lysosomal uptake	Altered gene expression, TGF-β dependent fibrosis pathway
Inflamatory events in light-exposed tissues	Apoptosis	Osteoporosis and vascular calcification, Bone deterioration
Increase, Clonal Expansion of Altered Hepatic Foci		Increased, adenomas (pituitary)
Increased, inflammation		Promotion, ovarian adenomas
systemic inflammatory response syndrome		Binding to ACE2
		Inflamatory events in light-exposed tissues
		Increased Pro-inflammatory mediators
		systemic inflammation leading to hepatic steatosis
		Unfolded Protein Response
		Suppression, Inflammatory cytokines
		Inhibition, Nuclear factor kappa B (NF-kB)

THP1-AO predicted	Lung-AO predicted	BEAS-2B-AO predicted
Inflamatory events in light-exposed tissues	Increased, adenosquamous carcinomas of endometrium	Inflamatory events in light-exposed tissues
Increased, mesotheliomas	Inflamatory events in light-exposed tissues	Promotion, ovarian adenomas
N/A, Neurodegeneration	Cognitive Function, Decreased	Increased, adenomas (pituitary)
Hyperinflammation	Increased, adenomas (pituitary)	Increase, Increased susceptibility to infection
N/A, Breast Cancer	Apoptosis	Hyperinflammation
Neural tube defects	Liver Injury	Decrease, Growth
Cholestasis, Pathology	Increased, Liver Steatosis	Ororofacial clefting
Increase, DNA damage	Increase, Site of Contact Nasal Tumors	N/A, Breast Cancer
Increase, Mutations	Increased, mesotheliomas	Neurodegeneration
Increased, Liver Steatosis	Increase, lung cancer	Neural tube defects
N/A, Liver fibrosis	N/A, Liver fibrosis	Increased, mesotheliomas
Increased, Ductal Hyperplasia	N/A, Breast Cancer	Apoptosis
Decrease, Lung function	Accumulation, Liver lipid	Immune mediated hepatitis
Accumulation, Liver lipid	Occurrence, Kidney toxicity	Parkinsonian motor deficits
	Parkinsonian motor deficits	Increase, Mutations
	N/A, Neurodegeneration	N/A, Neurodegeneration
		Decrease, Lung function
		Increased, Liver Steatosis
		Increased Mortality

THP1-MIE predicted	Lung-MIE predicted	BEAS-2B-MIE predicted
systemic inflammation leading to hepatic steatosis	systemic inflammation leading to hepatic steatosis	systemic inflammation leading to hepatic steatosis
ROS generation from photoactivated chemicals	Endocytotic lysosomal uptake	Activation, Glucocorticoid Receptor
Oxidative Stress	ROS generation from photoactivated chemicals	Oxidative Stress
Frustrated phagoytosis	Antagonism, Thyroid Receptor	Protein Adduct Formation
Alkylation, Protein	Activation, Androgen receptor	Alkylation, Protein
Inhibition, Bile Salt Export Pump (ABCB11)	Mitochondrial impairment	Covalent Binding, Protein
Activation, Glucocorticoid Receptor	narcosis	Activation, Estrogen receptor
Activation of Cyp2E1	Histone deacetylase inhibition	Histone deacetylase inhibition
Increase, DNA damage	Activation, PPARα	CYP2E1 Activation
Binding, Thiol/seleno-proteins involved in protection against oxidativ	Increase, Cytotoxicity (hepatocytes)	ROS generation from photoactivated chemicals
Activation, Estrogen receptor	Activation, AhR	Inhibition of N-linked glycosylation
Histone deacetylase inhibition	Direct mitochondrial inhibition	Frustrated phagoytosis
Increase in reactive oxygen and nitrogen species (RONS)	Increased, Hepatic thyroid hormone uptake/transport	Activation of Cyp2E1
Inhibition, IKK complex	Non-coding RNA expression profile alteration	Increase, DNA damage
Non-coding RNA expression profile alteration	Thyroperoxidase, Inhibition	Inhibition, IKK complex
Inhibition, NADH-ubiquinone oxidoreductase (complex I)	Thyroid hormone synthesis, Decreased	Increase in reactive oxygen and nitrogen species (RONS)
Thyroid hormone synthesis, Decreased	Increase, DNA damage	
Acetylcholinesterase (AchE) Inhibition	Acetylcholinesterase (AchE) Inhibition	
	Inhibition, Na+/I- symporter (NIS)	
	Decompartmentalization	

Table S2: Events enriched from the dataset GSE127773

Event	Padj	Description
Event 1512	5.09776232953353e-14	Unfolded Protein Response
Event:1825	6.19156257981582e-14	Increase, Cell death
Event:403	1.52814019037611e-13	Suppression, Immune system
Event:1817	2.44960766999024e-13	Apoptotic cell death
Event:1505	4.312678909358e-13	Cell cycle, disrupted
Event:1912	1.50327417433568e-12	Motile Cilia Number/Length, Decreased
Event:1183	2.93639390704635e-12	Decreased, Apoptosis (Epithelial Cells)

Table S3: Results of the clustering of the 93 co-expression networks. Netowkrs belonging to each cluster are reported in the same column.

Cluster 1	Cluster 2	Cluster 3	Cluster 4	Cluster 4
Beas2b_Tio2r_core	THP1_Tio2s_COOH	Lung_Ag_NH	THP1_Ag_NH	Lung_Au20_PEG
Beas2b_Au20_COOH	THP1_Tio2r_core	Lung_CuO_NH	THP1_MWCNT_core	Lung_ND_PEG
Beas2b_QD_COOH	THP1_ND_PEG	Lung_Tio2r_COOH	THP1_MWCNT_PEG	Lung_Ag_PEG
Beas2b_ND_NH	THP1_ND_COOH	Lung_Tio2r_core	THP1_ND_NH	Lung_Tio2s_PEG
Beas2b_Au5_NH	THP1_Tio2s_NH	Lung_CuO_COOH	THP1_Ag_COOH	Lung_ND_NH
Beas2b_CuO_COOH	THP1_Tio2s_core	Lung_CdTe_PEG	THP1_QD_NH	Lung_Au5_PEG
Beas2b_Ag_NH	THP1_CuO_PEG	Lung_CdTe_COOH	THP1_QD_PEG	Lung_Au5_COOH
Beas2b_MWCNT_COOH	THP1_Tio2r_NH	Beas2b_ND_PEG	THP1_Au5_NH	Lung_Tio2s_NH
Beas2b_Ag_COOH	THP1_Tio2r_PEG	Lung_CdTe_NH	THP1_CuO_COOH	Lung_Tio2s_COOH
Beas2b_Au5_COOH	THP1_Tio2r_COOH	Lung_MWCNT_COOH	THP1_Au5_COOH	THP1_Tio2s_PEG
Beas2b_CuO_PEG		Lung_MWCNT_NH	THP1_Au20_NH	Beas2b_Tio2s_PEG
Beas2b_Au20_PEG		Lung_CuO_core	THP1_MWCNT_COOH	Lung_Au20_NH
Beas2b_MWCNT_core		Lung_Tio2s_core	THP1_CuO_NH	Lung_ND_COOH
Beas2b_QD_PEG		Lung_Ag_COOH	THP1_Ag_PEG	Lung_Au20_COOH
Beas2b_Tio2s_NH		Lung_CuO_PEG	THP1_MWCNT_NH	Lung_Tio2r_PEG
Beas2b_CuO_NH		Lung_MWCNT_core	THP1_Au20_PEG	Lung_Au5_NH
Beas2b_Ag_PEG		Lung_MWCNT_PEG	THP1_Au20_COOH	
Beas2b_CuO_core		Lung_Tio2r_NH	THP1_CuO_core	
Beas2b_Au5_PEG			THP1_Au5_PEG	
Beas2b_Tio2r_COOH			THP1_QD_COOH	
Beas2b_Tio2s_core				
Beas2b_MWCNT_PEG				
Beas2b_Tio2r_PEG				
Beas2b_MWCNT_NH				
Beas2b_Tio2r_NH				
Beas2b_QD_NH				
Beas2b_ND_COOH				
Beas2b_Au20_NH				
Beas2b_Tio2s_COOH				
